# Stops making sense – For the people?

**DOI:** 10.1002/ctm2.1270

**Published:** 2023-05-18

**Authors:** Leoš Shivaya Valášek, Julius Lukeš, Zdeněk Paris

**Affiliations:** ^1^ Institute of Microbiology Czech Academy of Sciences Prague Czech Republic; ^2^ Institute of Parasitology Biology Centre Czech Academy of Sciences České Budějovice (Budweis) Czech Republic; ^3^ Faculty of Sciences University of South Bohemia České Budějovice (Budweis) Czech Republic

Dear Editor,

Thanks to the seminal work of Drs. Brenner, Jacob and Meselson from 1961, we have come to realize that our life depends on ‘an unstable intermediate (mRNA) carrying information from genes to microsomes (ribosomes) for protein synthesis’.^1^ Using the famous triplet‐binding assay with soluble S‐RNAs (transfer tRNAs) charged with radioactively labelled amino acids, Nirenberg and Leder completed in 1966 deciphering of the individual code words revealing the universal genetic code of all extant life.^2^ What have we learned since then about the universality of this omnipresent code? How could this knowledge contribute to improving our health? Let's take a look.

## 1 NONSTOP PROTISTS

The standard genetic code contains 64 codons, 61 of which (sense) code for specific amino acids and three define stop (nonsense) codons. The genetic code is universal; well, yes, as long as we accept the wise old adage that every exception proves the rule. Approximately 32 such exceptions have been documented so far, involving sense‐to‐sense, sense‐to‐stop and stop‐to‐sense alterations of the meaning of the genetic code.^3^ A few recently described ‘nonstop’ protists have turned out to be the most dramatic rule breakers known to date.^4^, ^5^, ^6^ Interestingly, whole genome sequencing revealed that all three stop codons (UGA, UAG and UAA) are dispersed in their coding sequences. Thus, for these organisms to survive, their nonsense codons must have acquired specific amino acid meaning.

**FIGURE 1:**
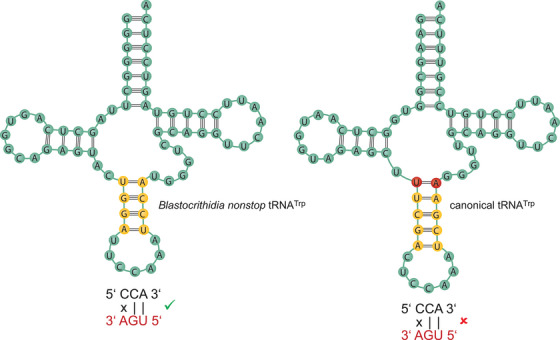
Unique, one base shorter anticodon stem of tRNA^Trp^_CCA_ is critical for UGA reassignment to tryptophan in a process called stop codon read‐through in *Blastocrithidia nonstop*. *Source*: Adapted with permission from magazine Vesmír – vesmir.cz.

Taking one of these protists, specifically the trypanosomatid flagellate *Blastocrithidia nonstop*, under the laboratory spotlight, we have recently discovered that UAG and UAA stops were reassigned to glutamic acid in the simplest possible way – by acquiring new genes encoding tRNAs^Glu^ with their anticodons fully cognate to these stop codons.^7^ The UGA reassignment to tryptophan occurred in a much more convoluted but at the same time fascinating way. We found that a relatively minor change in the structure of the *B. nonstop* tRNA^Trp^ (Figure 1), specifically the shortening of its anticodon stem (AS) from the canonical 5 to 4 bp, greatly enhances its ability to wobble base pair (i.e. pair with two of the three bases) with UGA. This unique alteration among eukaryotes, combined with a specific single amino acid substitution in the release factor eRF1 severely compromising its ability to decode UGA, then allows this non‐canonical tRNA^Trp^ to be more readily accepted by the ‘terminating’ ribosome (i.e. having UGA in its A‐site) in a process called stop codon read‐through (SC‐RT).^7^ Interestingly, our unpublished data suggest that similar small changes in the AS of other so‐called near‐cognate (nc) tRNAs, whose anticodons can establish two out of three canonical base pairs with some of the three stops, can achieve the same thing. Is this worth further investigation beyond our natural curiosity?

## 2 PREMATURELY STOPPED PATIENTS

Considering the ever‐growing list of genetically inherited, mostly rare and serious diseases, caused by incomplete synthesis of a given protein necessary for normal development and/or growth, we argue that findings derived from these rather obscure organisms may be relevant for human medicine. After all, the synthesis of these proteins cannot be completed due to the spontaneous occurrence of a pathogenic premature stop codon (PTC) in the coding sequence of its mRNA (for review see Refs. [^8^, ^9^]). The most (in)famous among them is cystic fibrosis (CF) causing severe damage to the lungs, digestive system and other organs, with a plethora of nonsense mutations in the CF transmembrane conductance regulator. Another serious ailment is Duchenne muscular dystrophy (DMD) that primarily affects young boys and is caused by mutations, including PTCs, of the dystrophin gene, the product of which ensures the structural integrity of muscle cells. Patients suffering from retinitis pigmentosa carry PTCs in the *FAM161A* gene, while those diagnosed with Shwachman–Diamond syndrome, which primarily affects the pancreas and bone marrow, struggle due to PTCs present in the *SBDS* gene. Junctional epidermolysis bullosa is caused by incomplete expression of COL17 in human keratinocytes due to a PTC. Human mucopolysaccharidosis type I (MPS I) is caused by the lack of α‐l‐iduronidase enzymatic activity due to PTCs in the *Idua* gene, triggering detrimental accumulation of glycosaminoglycans in lysosomes; and the list goes on. Indeed, nonsense mutations account for ∼12% of all known pathogenic mutations. The simplest cure for patients suffering from PTC‐caused diseases would thus be a targeted induction of SC‐RT on a ‘tiny’ but pathological PTC mutation, which is ‘hugely’ detrimental to their quality of life.

## 3 THE DRAWBACKS OF CURRENT PTC‐RELATED THERAPIES

The latter suggestion is obviously nothing new. Researchers have been trying to induce SC‐RT on PTCs by either increasing the incorporation rates of nc‐tRNAs and/or lowering translational accuracy using various read‐through‐inducing drugs (RTIDs) (for review see Ref. [^10^]). One class of RTIDs encompasses notorious aminoglycoside antibiotics, which directly bind to the ribosomal decoding centre and facilitate nc‐tRNA mispairing, thereby impairing translational accuracy. The other class features for example ataluren, which interferes with the activity of release factors. Expectedly, however, the non‐targeted effects of RTIDs on translation fidelity have undesirable consequences. These include increased SC‐RT of genuine stop codons, producing a wide range of proteins with C‐terminal extensions, as well as the so‐called misreading, which is caused by the incorporation of nc‐tRNAs at inappropriate codons that may severely compromise protein's integrity. As a result, patients enrolled in RTID‐based clinical trials suffer from serious side effects, such as hearing loss, balance disorders and so on. Despite the undeniable progress and hope that RTIDs have brought to the world of clinical research, it now appears that the lack of specificity, significant side effects and disappointing efficacy, as documented by already two unsuccessfully completed Phase III clinical trials of ataluren in the CF patients,^11^ have caused researchers to slowly step on the brakes of this research direction and search for other solutions.

## 4 COULD SPECIFICALLY MODIFIED READ‐THROUGH‐INDUCING tRNAs HELP?

**FIGURE 2:**
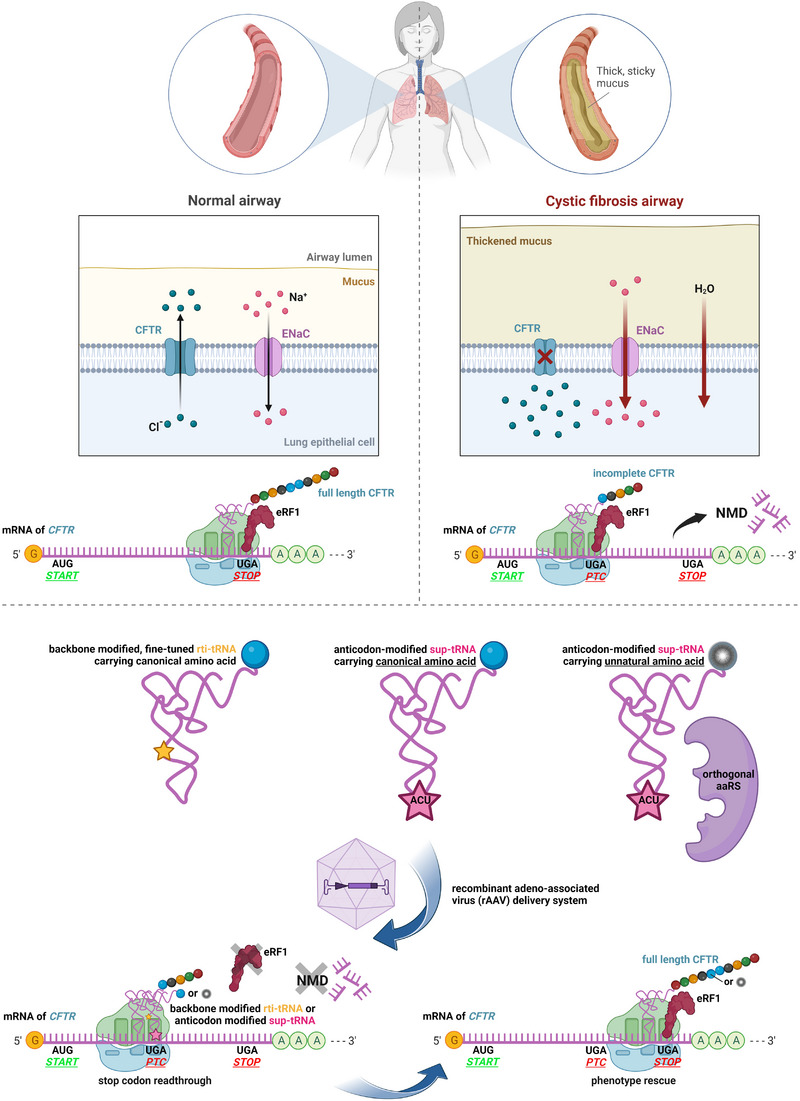
Potential of a modified tRNA molecule to restore synthesis of a full‐length protein such as cystic fibrosis transmembrane conductance regulator (CFTR) and rescue PTC‐caused diseases such as cystic fibrosis. *Source*: Created with BioRender.com.

It is tempting to think so. The read‐through‐inducing nc‐tRNAs (rti‐tRNAs) are natural to every organism, which means they should be well‐tolerated by our immune system, unlike the gene‐editing machineries such as the CRISPR‐based system. They are stop codon‐specific with tunable function, unlike RTIDs with almost random, non‐customizable incorporation of a given amino acid at the PTC that can compromise protein function and confer a dominant‐negative effect. Therefore, no dramatic adverse effects should be expected. All this makes this category of tRNAs well suited for personalized medicine with the promise of long‐lasting effects. The key questions that need to be addressed to pave the new road of the PTC‐oriented clinical research with tRNAs are: (1) their sustained and stable expression, effective charging and recruitment to the ribosomal A‐site; (2) their targeted and efficient delivery; (3) nonsense‐mediated decay pathway (NMD); (4) read‐through on genuine stops and (5) choice of a tRNA system (summarized in Figure 2). Unsurprisingly, each of these issues is not trivial, as detailed in the following.

Ad 1) The U6 promoter seems most suitable.^12^, ^13^, ^14^, ^15^ Effective tRNA charging and recruitment are determined by aminoacyl tRNA synthetases (aaRS) and elongation factor eEF1α, which recognize specific tRNA identity elements but not always the stop codon, therefore special care must be taken to fine‐tune the prospective therapeutic tRNAs.^16^, ^17^

Ad 2) Two recent studies have successfully pioneered a delivery route via a recombinant adeno‐associated virus (rAAV). The rAAV delivery of a suppressor tRNA safely and efficiently rescued the aforementioned human MPS I genetic disease in a mouse model carrying a nonsense mutation in the *Idua* gene.^14^ The same route combined with intraperitoneal administration of the specific unnatural amino acid was used to deliver the engineered tRNA‐synthetase–tRNA pair (PylRS – tRNAPyl UUA orthogonal system) in the DMD mouse model where it partial restored expression of dystrophin and recovered the muscle function.^15^

Ad 3) It is well known that efficient SC‐RT read‐through of a PTC can antagonize NMD of a PTC‐containing transcript during the first round of translation, as was recently further demonstrated in the mouse MPS I model.^14^

Ad 4) A major concern for all read‐through‐inducing tools is their impact on genuine termination codons throughout the transcriptome; however, a recent study has made us hopeful that ‘going‐the‐tRNA‐way’ is very promising.^14^

Ad 5) The selection from which to choose represents: A/ backbone modified, fine‐tuned rti‐tRNAs with the sequence and modification of the key anticodon loop preserved^7^, ^12^; B/ suppressor tRNAs derived from natural tRNAs by mutating their anticodon to perfectly match a given stop codon. The latter can carry either unnatural amino acids via the orthogonal aaRS‐tRNA pair systems^15^ or natural ones.^14^ In addition, either of these options may be coupled with a concomitant impairment of the function of both release factors,^18^ but this is of serious concern, particularly in view of the previous point.

We believe that further inspiration for how to deal with human diseases (not only those caused by PTCs) may come from organisms with non‐canonical genetic codes.^4^, ^5^, ^6^, ^7^ These organisms, for one reason or another, usually lost in their evolutionary history, have opted to deviate from the undeniably well‐tuned universal genetic code. They have had to bear the consequences of this decision, and in order to survive, have been forced to come up with ingenious solutions, facilitated by innumerable attempts over long evolutionary time scales. Only future research will tell how much we can learn from them for the benefit of our own health.

## CONFLICT OF INTEREST STATEMENT

The authors declare they have no conflict of interest.
